# A Smartphone Resource for Just-in-Time Medical Student Teaching by Emergency Medicine Residents

**DOI:** 10.7759/cureus.70117

**Published:** 2024-09-24

**Authors:** Joshua Ginsburg, Margaret Sande, Azhar Ahmed, James Moak, Amita Sudhir, Mary K Mutter

**Affiliations:** 1 Emergency Medicine, University of Texas Southwestern Medical Center, Dallas, USA; 2 Emergency Medicine, University of Virginia School of Medicine, Charlottesville, USA; 3 Emergency Medicine, Sentara RMH Emergency Department, Harrisonburg, USA

**Keywords:** emergency medicine research, just-in-time training, : medical education, medical student training, residents as teachers, technology enhanced education

## Abstract

Objectives

Previous studies have shown that residents play an important role in the education of medical students in the clinical setting, but the busy environment of the emergency department (ED) poses a challenge to the effective teaching of medical students by emergency medicine (EM) residents. To combat this, this study relies on just-in-time teaching, which refers to the application of teaching efforts specific to a particular moment, in this case, a clinical case or procedure. Building on studies showing that just-in-time teaching can improve education despite time constraints, as well as data showing that practitioners and trainees increasingly use apps for diagnosis and education, the objective of this project was to determine whether a free open-access medical education (FOAM) smartphone application (app) is feasible and improves the quality and frequency of resident teaching of medical students in the emergency department.

Methods

We created a smartphone app-based teaching resource that was available to residents and students on shift. We taught residents and medical students how to integrate the app into their workflow. We performed an observational pre/post-study design by surveying residents' and students' pre- and post-teaching app introduction to determine self- and student-reported teaching frequency and quality. Outcome measures were self-reported teaching frequency and quality of teaching.

Results

Ten of 17 residents (59%) reported using the app at least once per shift. Among them, six of 10 (60%) reported that it increased their teaching frequency, and eight of 10 (80%) reported that it increased the quality of their teaching. There was an increase in the number of residents with positive perceptions of the availability of evidence-based teaching resources on the shift from pre-intervention (five residents) to post-intervention (14 residents, p = 0.01); however, we were otherwise unable to identify any significant differences in residents’ or students’ perceptions of teaching after implementation of our intervention.

Conclusion

Just-in-time teaching of medical students by residents using a smartphone app is feasible, as evidenced by the majority of resident respondents reporting that they used the teaching app at least once per shift. Residents reported an improvement in teaching frequency and quality. The study is limited by a small sample size and a single-site design with a pre/post design. Further studies with a focus on qualitative feedback and objective measures of learning are needed to assess whether there is an impact on medical student instruction by residents when using this app-based teaching resource.

## Introduction

The Liaison Committee on Medical Education and the Accreditation Council for Graduate Medical Education have emphasized the importance of residents as teachers [1,2]. In previous studies, medical students have reported that up to one-third of their knowledge was attributable to resident teaching [3,4]. However, time constraints and heavy workloads make real-time bedside teaching in the emergency department (ED) challenging. Lack of time and a perception of increased workload have been identified as barriers to effective teaching [5]. Courses, skills sessions, and web-based tools to improve resident teaching have been proposed as means to improve resident teaching, but none reduce the amount of time required for actual bedside teaching [6-8].

Just-in-time (JIT) teaching for learners in a busy clinical environment is a well-researched teaching method that implements technological resources for learning immediately adjacent to the application [9-13]. The teaching method relies on a predefined, brief educational review, sometimes using simulation or a demonstration video before the learner undertakes a particular task. In previous studies, learners have reported that this method works well for education in a time-constrained learning environment [9]. Although prior interventions to improve JIT teaching have focused primarily on procedural skills education, JIT teaching has also shown promise for improving the quality of core content education [14]. We, therefore, hypothesized that the implementation of a JIT teaching smartphone application (app) using curated educational content from free and open access medical education (FOAM) sites about core topics, decision rules, and procedures would reduce residents’ time burden for teaching and improve the quality of their teaching. 

While healthcare practitioners and trainees use apps for diagnosis, management, and drug referencing, and medical students frequently use smartphones for medical education, this study addresses the real-time use of a content-based app employing JIT teaching to improve the quality and frequency of teaching in the ED [15,16]. Our study objective was to determine whether a FOAM-based smartphone app is feasible and improves the frequency and quality of resident JIT teaching bedside education of medical students.

## Materials and methods

Curricular design

Based on the goals, objectives, and core content of our institution’s emergency medicine (EM) third-year clerkship, seven broad topics were identified. Two EM residents (JG, AA) with an interest in medical education then created portable document format (pdf) files about these seven core EM topics: headache, chest pain, shortness of breath, abdominal pain, psychiatric illnesses, procedures, and express care complaints. Each file was reviewed by a resident volunteer and one of three EM faculty (MKM, AS, JM) with education leadership roles within the department. Each file contained an approach to the chief complaint or topic, associated differential diagnosis, recommended workups, treatment options, and more detailed information organized by diagnosis. Under each diagnosis were links to free open-access medical education (FOAM) articles, calculators, and other evidence-based external links. We aggregated our files into the mobile app Yapp (Yapp Inc., New York, United States), which was chosen for ease of implementation, as it was required by the medical school to be downloaded onto all medical students' cellular phones to organize their clerkship materials.

We attempted to spread awareness of this new educational resource through three distinct, multimodal means. First, we sent emails to the EM residents with instructions on how to download the app and suggestions regarding the best use of the educational resource file to engage the medical students in JIT teaching. To serve as a more constant reminder, we additionally posted laminated cards on each ED computer with the same information. Finally, for an in-person explanation, we presented the project during the weekly resident education conference to show residents ideal ways to use the app for their JIT teaching. We proposed but did not limit residents to three ways to use the resource file: student-selected, case-generated, and oral case presentation-generated. An oral case presentation is the history, physical exam, assessment, and plan as described to the resident or attendee. For the student-selected method, we recommended residents ask students what they would like to focus on for the day and then direct them to an appropriate resource to read and discuss between patients. For the case-generated method, residents were encouraged to direct students to a topic or link to read after seeing a patient in order to generate clinical questions and help prepare the student for the oral case presentation to the attending. Finally, for the oral case presentation generated method, the resident was advised to decide on a topic or link for students to explore after their patient oral case presentations to the attending or resident. Residents were given the freedom to choose the method they felt was most appropriate for a given case, and we did not collect data on which method was chosen.

Statistical methods

This was an observational pre/post study, performed over the course of six months at the University of Virginia (UVA) in Charlottesville, Virginia. The study was exempted by the UVA Institutional Review Board (protocol number 5401). The UVA Emergency Medicine Residency has 36 residents over a three-year program, and the School of Medicine has approximately 150 medical students per class. All students are required to rotate through an emergency medicine clerkship.

We created a six-question pre-intervention survey on a five-point Likert scale ranging from “strongly disagree” to “strongly agree” with questions about residents’ perceived frequency and quality of their own teaching as well as their perceived availability of evidence-based teaching resources (Appendix 1). The survey was piloted with three EM residents (10% of the study population) to refine the clarity of language and ease of use. The survey was administered toward the end of the academic year when all residents had rotated through the ED several times and would have had ample teaching opportunities. All residents were invited by email to participate in the pre-survey. Six months later, we administered the same survey to second and third-year residents, with an additional three questions assessing the use of the resource files (Appendix 2). After weighing their likely inability to provide meaningful survey responses against the potential introduction of bias, we elected to exclude first-year residents from the post-intervention survey because many of them had been on off-service rotations with few teaching opportunities in the ED. The sample size of our cohort was determined by available residents. Written consent was obtained from all residents. We also created a separate six-question survey on a five-point Likert scale to gauge medical students’ perceptions of the frequency and quality of evidence-based teaching by emergency medicine residents (Appendix 3). The survey was piloted with a small focus group of medical students (2% of the study population) to refine the clarity of language and ease of use. The survey was administered to 75 students at the end of their three-week clerkship for several months before and 57 students after the implementation of our innovation. Written consent was obtained from all students. Resident and student responses to the pre-intervention and post-intervention survey questions were summarized by frequency distributions and analyzed by logistic regression. Tests of statistical inference compared the odds for a positive response to the survey question (i.e., “agree” or “strongly agree”) from pre-intervention to post-intervention.

## Results

Resident survey results

Nineteen of 30 eligible residents (63%) participated in the pre-intervention survey, and 17 of 22 (77%) participated in the post-intervention survey. Of these 17 residents, 10 residents (59%) reported using the resource file at least once per shift. Six of those 10 residents (60%) reported that it increased their teaching frequency, and eight of those 10 residents (80%) reported that it increased the quality of their teaching.

**Figure 1:**
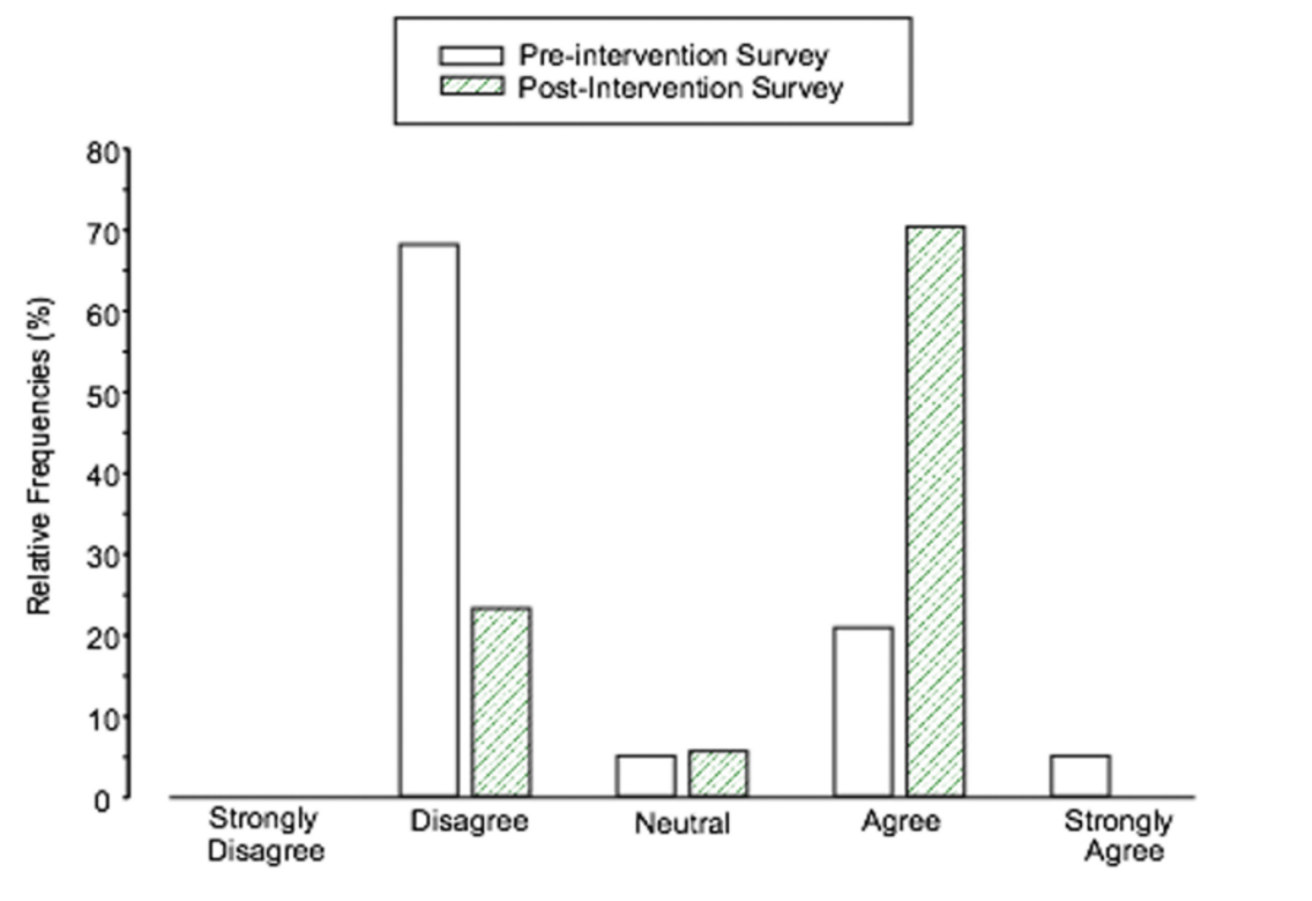
Relative frequency distribution for the pre-intervention and post-intervention responses to “I have adequate evidence-based resources readily accessible during shifts to facilitate teaching.”

We found a statistically significant increase in the number of residents with positive perceptions of the availability of evidence-based teaching resources on the shift from pre-intervention (five) to post-intervention (12), p = 0.01 (Figure 1). For the remainder of the questions, we found no statistically significant differences.

Medical student survey results

Seventy-five medical students participated in the pre-intervention survey, and 57 participated in the post-intervention survey. There was no statistically significant change in medical student responses to the individual questions from pre- to post-intervention.

## Discussion

Our findings show that a FOAM-based smartphone app may have a role in improving just-in-time teaching of medical students by residents in the busy environment of the emergency department. Of the EM residents who used the FOAM-based smartphone app, a significant percentage reported that it increased their teaching frequency and quality. There was also a statistically significant improvement in resident perceptions of evidence-based teaching resources. These findings indicate not only that the use of the app during a busy ED shift is feasible, but also that it may contribute a meaningful evidence-based teaching resource to improve teaching frequency and quality.

Though we were encouraged by these initial positive findings from our analysis, we were unable to demonstrate a clear, significant positive impact on student education based on students’ perceptions of teaching quality and frequency. Anecdotal student and resident feedback was positive, and students shared with study leaders that they found the resource to be very helpful. Students independently notified the subsequent student groups about the resource’s availability. However, 40% of residents reported they did not use the resource at least once per shift, and even among those who did, several reported that it did not increase the frequency or quality of their teaching. One possible explanation for this discrepancy is that we did not achieve successful diffusion of innovation. The diffusion of innovation theory explains how, why, and at what rate new technologies spread. Inadequate adoption of an innovation could be explained by the innovation’s attributes, the characteristics of the adopters, and the larger social context [17]. Specifically, we believe that the negative findings of our study may have been the competing needs of residents and medical students. While the app was designed to provide students easy access to curated content, this might not have saved residents time in their workflow during a busy shift. Overcoming this hurdle of utilizing a new tool and troubleshooting it alongside a student may have been an entry barrier when compared to a resident’s established teaching behaviors. For future iterations of this project, it may be impactful to use a design thinking approach by making observations of how residents function in their environment and seeking more widespread input from both mainstay users and outliers. Interestingly, a similar app-based educational intervention for pediatric resident clinical teaching had a similar implementation percentage, with 60% of residents using the app in at least some capacity [18]. We were not able to obtain more detailed feedback from residents about what made the app easy or difficult to integrate into the resident workflow, as residents did not respond to follow-up feedback. Important next steps for this project are to obtain qualitative feedback about the app’s particular positive and negative attributes through surveys, interviews, or in-app feedback functions, and to better understand the particularities of the emergency medicine residents’ barriers to teaching [19].

Our findings must be interpreted in light of several limitations. First, our sample size for resident participants was small, as we were limited by the number of residents in our program. For this reason, no power analysis was performed. Additionally, our survey instrument was created by a process of development, feedback, and refinement but was not based on a previously validated survey. Our survey piloting was robust as a percentage of residents (10%), but more limited as a percentage of medical students (2%). The resident response rate was relatively low, with 63% of residents responding to the pre-intervention survey and 77% responding to the post-intervention survey, which may introduce non-response bias. Additionally, the difference in the number of medical student participants from pre- to post-intervention may have affected the reliability of the comparative analysis. Our study relied on a potentially inaccurate self-report of app use, as the chosen app did not have a function to track use. We also relied on self-reporting of teaching frequency and teaching quality due to the difficulty of using objective, quantifiable measures of these outcomes in the ED. Finally, with an observational design study, we could not account for changing educational factors over time nor for variability among the pre- and post-characteristics of both residents and students over the study period

## Conclusions

This study suggests that a FOAM-based smartphone app may have a role in improving evidence-based JIT medical student teaching by residents in the ED. Residents perceived a greater availability of evidence-based teaching resources on shift, and, among those who used the app, increased frequency and quality of teaching. Future investigations may benefit from a larger sample size, qualitative feedback about the benefits and challenges of the app, as well as more objective measures of app use, teaching frequency, and teaching quality.

## Appendices

Appendix 1

Resident Survey

1.    I am satisfied with the frequency with which I teach during my shifts. 1 = strongly disagree, 2 = disagree, 3 = neither agree nor disagree, 4 = agree, 5 = strongly agree

2.    I am satisfied with the quality of my teaching during my shifts. 1 = strongly disagree, 2 = disagree, 3 = neither agree nor disagree, 4 = agree, 5 = strongly agree

3.    I am able to teach medical students while taking care of patients. 1 = strongly disagree, 2 = disagree, 3 = neither agree nor disagree, 4 = agree, 5 = strongly agree

4.    I have an adequate understanding of what is expected for a third- or fourth-year medical student to learn during their EM clerkship. 1 = strongly disagree, 2 = disagree, 3 = neither agree nor disagree, 4 = agree, 5 = strongly agree

5.    I have adequate evidence-based resources readily accessible during shifts to facilitate teaching. 1 = strongly disagree, 2 = disagree, 3 = neither agree nor disagree, 4 = agree, 5 = strongly agree

6.     I teach medical students about my patients using evidence-based resources. 1 = strongly disagree, 2 = disagree, 3 = neither agree nor disagree, 4 = agree, 5 = strongly agree

Appendix 2

Additional Post-intervention Questions on Resident Survey

7.    The teaching file improved the frequency of my teaching of medical students during my shifts. 1 = strongly disagree, 2 = disagree, 3 = neither agree nor disagree, 4 = agree, 5 = strongly agree

8.    The teaching file improved the quality of my teaching of medical students during my shifts. 1 = strongly disagree, 2 = disagree, 3 = neither agree nor disagree, 4 = agree, 5 = strongly agree

9.     I used the teaching file to teach medical students on my shifts: ●    Never ●    Once per shift ●    2-5 times per shift ●    5-8 times per shift ●    >8 times per shift

Appendix 3

*Student Survey* 1.    I was satisfied with the frequency of resident teaching during my shifts. 1 = strongly disagree, 2 = disagree, 3 = neither agree nor disagree, 4 = agree, 5 = strongly agree

2.    I was satisfied with the quality of resident teaching during my shifts. 1 = strongly disagree, 2 = disagree, 3 = neither agree nor disagree, 4 = agree, 5 = strongly agree

3.    The residents I worked with as a whole seemed interested in teaching. 1 = strongly disagree, 2 = disagree, 3 = neither agree nor disagree, 4 = agree, 5 = strongly agree

4.    The residents I worked with taught me clinical pearls useful for learning the practice of emergency medicine. 1 = strongly disagree, 2 = disagree, 3 = neither agree nor disagree, 4 = agree, 5 = strongly agree

5.    There were adequate evidence-based resources readily accessible during shifts to facilitate learning. 1 = strongly disagree, 2 = disagree, 3 = neither agree nor disagree, 4 = agree, 5 = strongly agree

6.     The residents taught me about my patients using evidence-based resources. 1 = strongly disagree, 2 = disagree, 3 = neither agree nor disagree, 4 = agree, 5 = strongly agree
